# Promoting Survival and Primitive Reflexes to Prevent Brain Imbalance in Premature Infants: A Scoping Review of New Insights by Physiotherapists on Developmental Disorders

**DOI:** 10.7759/cureus.43757

**Published:** 2023-08-19

**Authors:** Pallavi Harjpal, Rakesh K Kovela, Moh'd Irshad Qureshi

**Affiliations:** 1 Neurophysiotherapy, Ravi Nair Physiotherapy College, Datta Meghe Institute of Higher Education and Research, Wardha, IND; 2 Physiotherapy, Nitte Institute of Physiotherapy, Nitte (Deemed to be University), Mangalore, IND

**Keywords:** scoping review, physiotherapy, premature infants, primitive reflex, neonatal intensive care unit, developmental disorders

## Abstract

Survival reflexes, originating from the brainstem, are involuntary motor responses that are present at birth and facilitate the survival of the neonate. The age of the baby is critical enough to give information about the maturation of these reflexes. In the case of preterm babies, the delayed maturity of these reflexes may pose a threat to the life of the newborn. One can perceive what the baby can feel, taste, smell, see, and hear through reflex maturation. The objective was to identify and understand the role of survival reflexes and primitive reflexes and their importance in premature children. PubMed, Cumulative Index to Nursing and Allied Health Literature (CINAHL), ProQuest, The Cochrane Library, Scopus, and Web of Science were the electronic databases used from January 2017 until November 2022. We included the original articles, reviews, and randomized clinical trials that focused on the importance of survival reflexes. Later on, all the articles were systematically arranged as per the information they provided, and 101 titles were selected, of which 32 met the inclusion criteria. Various articles were written regarding the present literature about primitive reflexes, but none promoted them in the neonatal intensive care unit (NICU).

This review is regarding the use of survival reflexes to improve the outcomes of neonates, specifically in the NICU. Simple interactions with the environment are made possible by primitive reflexes, which also serve as the foundation for early movement. This review presents a better understanding of the maturation of survival reflexes and primitive reflexes and provides further insight into how a physiotherapist can concentrate on the early identification and development of these reflexes to prevent further complications. Assessing the primitive reflex in the NICU will help in the early identification of developmental delay and further help us predict reflex maturation. Promoting them will provide positive outcomes in terms of neonatal development. A physiotherapist can play a vital role starting from the NICU to get the baby into an environment similar to the mother’s womb and therapy to get the early maturation of the reflex.

## Introduction and background

Survival reflexes, originating from the brainstem, are involuntary motor responses that are present at birth and facilitate the survival of the neonate [1]. The age of the baby is critical enough to give information about the maturation of these reflexes. In the case of preterm babies, the delayed maturity of these reflexes may pose a threat to the lives of newborns. Through reflex maturation, we learn about what the baby can feel, taste, smell, see, and hear. Premature babies are swaddled to provide a similar environment to that of a mother’s womb [2]. Premature babies tend to be attracted to sweetness. Likewise, the baby recognizes the mother's smell and tries to turn toward and reach that side. A baby is accustomed to the mother’s biorhythm and her timing for sleep and wake cycles; the effect of the constant lights in the neonatal intensive care unit (NICU) affects the same in premature babies [3]. Thus, by accessing these senses, we can learn about the reflexes' physiological maturity.

Survival reflexes play an important role in the neurological examination and management of the neonate. These reflexes are used to access the functional integrity of the basal ganglia, brainstem, and central nervous system. The reflexes that play the most important role in survival are the sucking, rooting, and snout reflexes [4]. The sucking reflex plays an important role in proper breathing and swallowing. When the oral region is stimulated or an instrument is inserted into the mouth at 14 weeks of pregnancy, it is initially noticed. The lips pucker as a result of pressure applied to the upper lip, causing the snout reflex. The rooting reaction can be seen as the patient's lips turning in the direction of an object when it is gently stroked on the cheek or placed in his or her field of vision. At 32 weeks of pregnancy, rooting begins, and over the subsequent month, it gradually decreases. The other reflexes are the asymmetric tonic neck reflex, symmetric tonic neck reflex, palmer reflex, plantar reflex, and Moro reflex. Most of these develop before birth during 28 weeks of gestation and disappear as soon as voluntary control is gained. They protect the newborn from different injuries.

Primitive reflexes are highly stereotyped responses generated by certain sensory stimuli. The sucking, Moro, and Babinski reflexes are among the primitive reflexes that have been extensively studied in the literature due to their significance. The sucking reflex, which works in tandem with breathing and swallowing to provide nutrients for life and growth, is vital for oral feeding. The Moro reflex is a naturally occurring protective motor reaction to abrupt shifts in bodily balance or intense stimulations. The Babinski reflex is a pyramidal tract nociceptive motor response that affects the foot's extensor and flexor muscles. Given the importance of these three reflexes for survival, defense, and development in newborns, understanding them may improve the neuro-behavioral assessment of the infant.

Prematurity exposes the baby to a very different environment in the NICU as compared to the one provided in the mother’s womb. This deprives the newborn of the mother-linked sensory inputs that are very essential during the crucial period of brain development and reflex maturation. Mostly, the problem arises from feeding itself; premature babies born before 32 weeks will have incoordination between sucking, swallowing, and breathing. In contrast, a term baby is habituated to sucking in the womb, and by 34 weeks, they develop coordination skills. If this is affected, then it will lead to life-threatening events of this coordination, not only affecting the feeding but also leading to aspiration, apnea, bradycardia, and hypoxia [2]. Feeding dysfunction affects the infant through the use of lots of energy, thus delaying its development [5].

The survival of high-risk neonates, including both full-term and preterm infants that required critical care, has significantly increased owing to medical innovations and improved neonatal care. In the NICU, doctors are seeing newborns who are smaller, more susceptible to illness and have extremely low birth weights (ELBW). It's crucial to monitor high-risk neonates' health from an early age because they have a higher mortality rate and are more likely to experience a range of health and developmental issues. In order to assess the baby's condition and share information with their families and other medical experts, multidisciplinary-based critical care in the NICU uses a wide range of examinations and multidisciplinary approaches to treatment.

Sucking reflexes in premature babies are often immature or weak, making it difficult for them to feed themselves. In reaction to pressure applied to the preemies' palms, the hand-to-mouth reflex, often referred to as the Babkin reflex, involves rotation and flexion of the head, typically with the mouth open. Preemies may suck their hands or fingers as a result of this basic instinct. The Babkin reflex is mediated by the brainstem's reticular formation, which also gets input from nonprimary motor cortices. More adaptive movement based on the hand-mouth reflex emerges when control of the nonprimary motor cortices over the reflex mechanism in the reticular formation becomes stronger. The voluntary eye-hand-mouth coordination needed for eating develops when the prefrontal cortex obtains control of the nonprimary motor cortices [6].

Now we discuss what a physiotherapist can do to prevent this delay and promote the health of premature infants. Usually, we provide oro-motor stimulation to develop sucking [7]. Along with this, we need to promote a motherly environment in the baby’s surroundings to ensure the overall development of the premature [8, 9]. This can be done by providing a warm, dim light environment, putting a piece of mother’s cloth in the vicinity for a sense of comfort, and making voice recordings of the mother, as this is what the baby is next to in the womb. This will not only help in reflex maturation but also in brain development, reducing hospitalization.

The fetus is exposed to a variety of patterns of vestibular, tactile, somatosensory, auditory, and visual stimulations in the intrauterine environment. Many of these stimulations are rhythmic, and the fetus picks up on these patterns and distinguishes between them. The fetus picks up on these diverse rhythms (the mother's heartbeat, breathing, speaking, and movement) and adjusts its behavior in reaction to them. Rhythmical vestibular stimulation is not available in the NICU.

Vestibular stimulation can be started right in the NICU. This vestibular rhythmic stimulation would complete the many rhythmic stimulations that the infant may get, which are all in sync with one another and, as a result, with the mother. Attachment development requires synchronous interactions in which both the parent and the preterm newborn are sensitive to one another. When evaluating the preterm infant's behavioral states, the mother's different sensory and rhythmic interventions have beneficial effects on development [10].

For physiotherapy and invasive procedures in the NICU, parent education and collaboration are essential. Kinesthetic stimulation helps to avoid neuromuscular disorders in neonates admitted to the NICU. Very preterm newborns benefit from interventions like kinesthetic stimulation and vestibular sensory systems. Swaddling and kangaroo care are two techniques that can help minimize discomfort and affect favorable neurobehavioral states. Early care treatments have a positive impact on the neurodevelopmental states of preterm neonates. Lack of exercise induces bone demineralization and development retardation in newborns due to protracted immobility. According to research, therapist-assisted range of motion exercises in multiple joints help premature infants gain weight and boost their bone mineral density.

## Review

Purpose

To identify and understand the role of survival reflexes and primitive reflexes and their importance in premature children.

Methods

We developed a search strategy using the Preferred Reporting Items for Systematic Reviews and Meta-analyses extension for Scoping Reviews (PRISMA-ScR) [11]. This comprehensive study answers a specific topic about the role of survival reflexes and their promotion in the NICU. Because the idea of promoting the health of immature babies through survival reflexes is relatively new, there hasn't been a scoping review of this subject to date. Utilizing the five-stage review framework recommended by Arksey and O'Malley, the data were gathered from electronic databases including Cumulative Index to Nursing and Allied Health Literature (CINAHL), PubMed, ProQuest, The Cochrane Library, Web of Science, and Scopus from the studies published from January 2017 until November 2022 [12]. We included original articles and randomized clinical trials (RCTs) that focused on the importance of survival reflexes. We used the following keywords for retrieving relevant studies: survival reflexes, primitive reflex, neonates, neonatal ICU, and physiotherapy. In order to identify studies that might be qualified, two review writers independently evaluated each study's title and/or abstract. Studies were acquired, and their eligibility was independently assessed. The third investigator will discuss any discrepancies between the three independent assessors. Eligibility criteria included studies that included patients in neonatal ICUs in the study setting of a NICU and studies that included RCTs and original articles.

**Figure 1:**
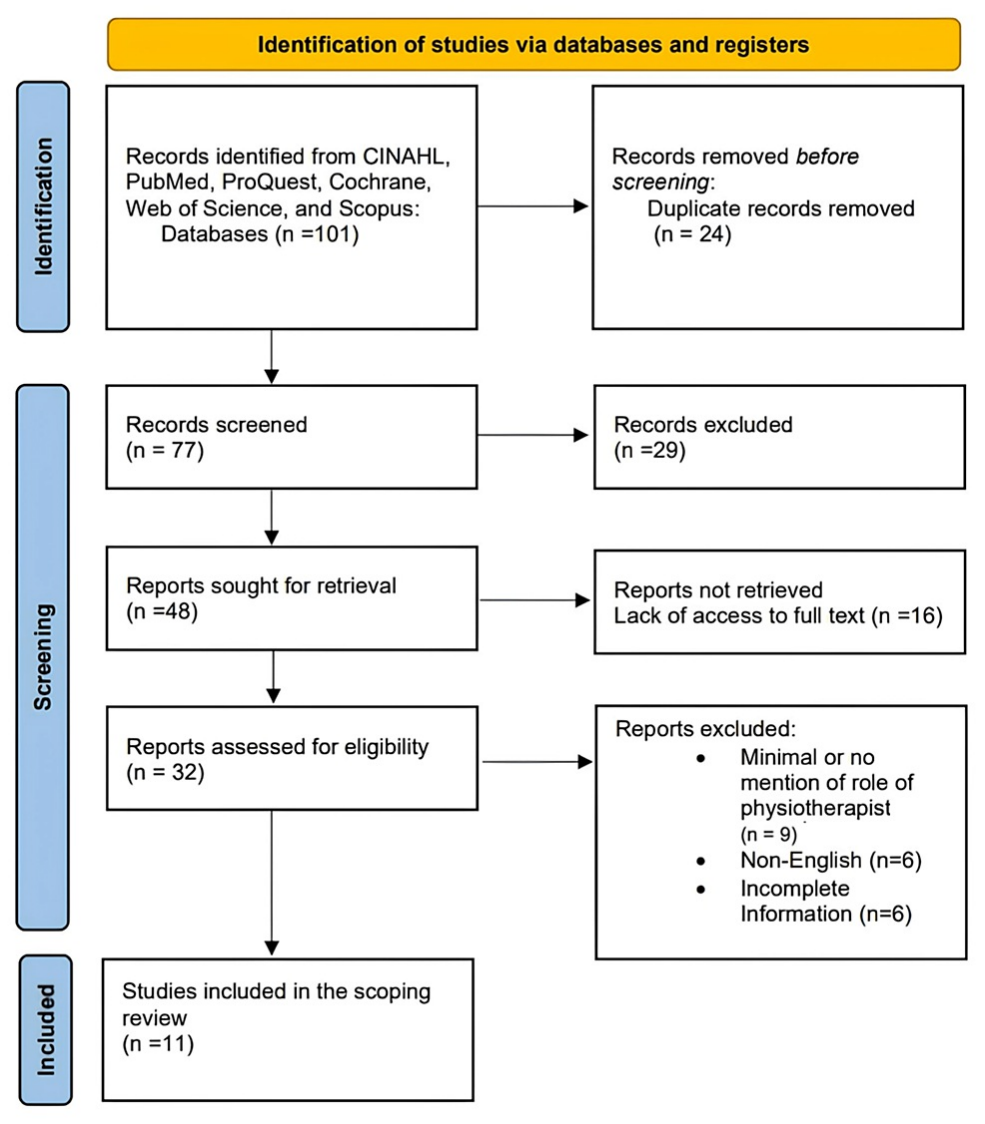
PRISMA flow diagram of the study CINAHL: Cumulative Index to Nursing and Allied Health Literature

Only human studies that have been published in peer-reviewed journals (peer-reviewed) full-text English papers were included in the search (Figure 1).

This proposed scoping review was carried out in accordance with the Preferred Reporting Items for Systematic Reviews and Meta-analyses extension for Scoping Reviews (PRISMA-ScR) [11] and the Joanna Briggs Institute (JBI) technique [13].

Result

One hundred and one titles were screened, and 32 met the inclusion criteria. Out of these, 11 were selected for review as the full text was available. Most of them were original review articles and randomized controlled trials (Level 1. c) [13]. Most of the articles were regarding the present literature about primitive reflexes, but none promote them in the NICU. This review is regarding the use of survival reflexes to improve the outcomes of patients in the NICU. The articles selected for the study are provided in Table 1.

**Table 1:** Matrix of the articles selected for review NICU: neonatal intensive care unit; CNS: central nervous system

S. No.	Title	Author, ref	Journal	Study design	Main finding	Author’s perspective
1	Feasibility and Impact of a Pilot Neonatal Cuddler Program on Preterm Infants in the Neonatal Intensive Care Unit	Bushroe et al., 2022 [14]	American Journal of Perinatology	Randomized controlled trial	In the neonatal intensive care unit, preterm infants deal with stress on a regular basis. Positive sensory experiences reduce stress reactions; however, parental involvement may be constrained by outside influences. The purpose of this study was to explain how a neonatal cuddler program (NCP) affected the growth of preterm newborns. Despite little interaction, an NCP has a good impact on growth.	The neonatal cuddler program has a positive impact on neonatal growth as it has the same effects as the vestibular stimulation provided by the movement of amniotic fluid in the mother’s womb. It leads to vestibular system maturation, providing positive effects on stress reduction. It also helps to gain a positive sensory experience when parents are not by the baby's side.
2	Comparison of the effect of two methods of sucking on the pacifier and the mother's finger on oral feeding behavior in preterm infants: a randomized clinical trial	Shaki et al., 2022 [15]	BMC Pediatrics	Randomized controlled clinical trial	Due to the positive effects of these two low-cost procedures, especially non-nutritive sucking on the mother's finger, on boosting oral eating behaviors rather than pacifiers, it is encouraged to employ them for preterm newborns admitted to neonatal critical care units.	Non-nutritive sucking promotes the development of the rooting and sucking reflexes that help to gain the coordination of sucking, swallowing, and breathing. It helps to develop proper oral feeding behavior in preterm infants.
3	Clinical effects of oral motor intervention combined with non-nutritive sucking on oral feeding in preterm infants with dysphagia	Li et al., 2022 [16]	Jornal de Pediatria	Randomized controlled trial	Oral motor intervention and non-nutritive sucking can significantly increase a premature neonate's oral feeding capacity, which also helps to facilitate oral feeding and reduces the chance of adverse effects. The combination intervention appears to improve preterm infants' oral feeding competence.	Infants who have trouble eating have trouble controlling their breathing, sucking, and swallowing. The oral motor intervention and non-nutritive sucking have beneficial impacts on enhancing eating abilities, as this aids in gaining control over the sucking reflex.
4	Neonatal aquatic physiotherapy in neonatal intensive care units: A scoping review	Aranha et al., 2021 [17]	Journal of Neonatal-Perinatal Medicine	Scoping review	Neonatal aquatic physiotherapy may be a useful method for NICU-admitted neonates to improve muscle tone, promote active movement, improve postural organization, improve sleep patterns and quality, and lessen neonatal pain.	Aquatic therapy for neonates helps to create an environment similar to the mother's womb, providing vestibular and somatosensory stimulation, improving muscle tone, and reducing neonatal pain.
5	Early combined rehabilitation intervention to improve the short-term prognosis of premature infants	Liu et al., 2021 [18]	BMC Pediatrics	Interventional study	Premature infants' short-term clinical outcomes can be improved with early integrated rehabilitative intervention.	Premature infants who receive early intervention right from the neonatal ICU have a positive outcome in later life, as most of the complications of prematurity can be overcome with early intervention.
6	Effectiveness of Oral Sensory-Motor Stimulation in Premature Infants in the Neonatal Intensive Care Unit (NICU) Systematic Review	Gonzalez et al., 2021 [19]	Children	Systematic review	By reducing the days of admission and the time it takes to achieve full oral feeding, oral sensorimotor stimulation helps preterm infants learn to feed themselves independently.	Oral motor stimulation promotes the development and maturation of survival reflexes, preventing complications of prematurity and reducing hospital stays.
7	Primitive Reflex	Alexa K. Modrell and Prasanna Tadi, 2021 [20]	Primitive Reflexes, PubMed (nih.gov)	Review	Neonatal life depends on primitive reflexes, and aberrant reflexes could be a sign of a failing central nervous system. For the early identification of potentially life-threatening issues, it is crucial to comprehend how to appropriately elicit these responses. Primitive reflexes aid in the diagnosis and treatment of neurological conditions.	Primitive reflexes are the survival reflexes that indicate CNS maturation. Early detection of abnormalities prevents complications and improves management.
8	Rooting Reflex	Hannah Yoo and Dana M. Mihaila, 2021 [4]	Rooting Reflexes, PubMed (nih.gov)	Review	Although the rooting reflex normally fades around four to 6 months, its persistence beyond this point could indicate prenatal cerebral injury. The frontal brain controls the rooting reflex, which can display a range of reactive abnormalities.	The rooting reflex is essential for survival, as its persistence indicates a CNS injury. It helps to gain gaze control, vestibular system maturation, and sucking control.
9	Early vocal contact and music in the NICU: new insights into preventive interventions	M. Filippa et al., 2020 [8]	Pediatrics research	Review article	The neuroscientific foundations of early perception, as well as the long-term impacts of music and early voice contact on the development of premature newborns, must be established.	Early vocal contact helps to develop the parent-newborn bond in the early stages of development. As it is rightly said "Mother’s heartbeat is the most pleasant sound for the baby", music therapy has the same calming effect as the mother’s heart sound the baby hears in utero.
10	The effects of premature infant oral motor intervention (PIOMI) on oral feeding of preterm infants: a randomized clinical trial	Ghomi et al. 2019 [5]	International Journal of Paediatric Otorhinolaryngology	Randomized clinical trial	Before the control group, the intervention group had their first oral feeding (on average, 7.2 days) and eight oral feedings (on average, 13.47 days). The results demonstrate the efficacy of PIOMI in the management of preterm newborns.	PIOMI improves the feeding of neonates by improving the survival response of the newborn. Weight gain is promoted with PIOMI and helps preterm infants with low birth weight.
11	Occupational therapy, physical therapy and speech-language pathology in neonatal intensive care unit: Patterns of therapy usage in NICU	Ross et al., 2017 [21]	Research in Developmental Disabilities	Interventional study	To improve outcomes, early therapy sessions in the NICU might begin during pregnancy and continue until NICU discharge.	An integrative approach in the neonatal intensive care unit helps in the early development of premature infants and prevents complications.

Discussion

The proposed review provides a better understanding of the maturation of survival reflexes and prevents further complications. Primitive reflexes are involuntary motor responses that start in the spinal cord and brainstem and aid in the neonate's survival. The environment that the baby gets in the mother's womb helps in the growth of vestibular, olfactory, gustatory, auditory, and visual systems that help the baby develop reflexes that later on turn into voluntary actions and help in development.

The infant nervous system has a high degree of plasticity during the first six months after birth, which is also the period of maximum brain growth and development [22]. The brain's ability to change, specifically through learning and compensating, is the foundation of early intervention. The environment easily affects the brain's ability to adapt and remodel its structure and function [23]. As a result, the newborn can quickly begin to actively recuperate during this time [24]. The prognosis of premature newborns can be improved by early intervention [25].

As per M Filippa et al., activity-dependent plasticity modifies the fetal brain throughout the last trimester of pregnancy, and preterm has been demonstrated to affect conventional developmental trajectories. Preventive therapies aimed at regulating these developmental trajectories through activity-inducing interventions are currently being investigated during this crucial period [8]. Similarly, according to Ross et al., occupational therapy and physiotherapy can be started right from the NICU to get an early positive outcome and prevent further complications during development [21]. Liu et al., in their study, suggested that premature infants' short-term clinical outcomes can be improved with early integrated rehabilitative intervention [18].

Graven suggested that infants should have plenty of opportunities to hear their parents' voices while they are talking to each other at the bedside. The atmosphere that should be supplied will safeguard sleep, promote steady vital signs, enhance the infant's speech comprehension, and lessen any potential negative impacts on auditory development [26].

The complicated coordination of breathing, swallowing, and sucking is necessary for oral feeding. While breathing may not fully integrate with sucking and swallowing until 34-42 weeks, sucking and swallowing normally reach maturity around 32 weeks [27,28]. Preterm newborns frequently experience delays in the development of their oral feeding and motor abilities because they don't get the same in-utero oral motor practice that comes from swallowing amniotic fluid [28]. Because of their underdeveloped reflexes, reduced muscle tone around the mouth, and diminished sensitivity and strength in their tongue, preterm newborns frequently have impaired oral motor control [19,29].

Touch therapy and oral function training can promote body growth, the development of gastrointestinal function and neuromotor function, the coordination of neuromotor function, and an increase in the frequency of bowel movements in preterm infants to improve their prognosis and ability to feed themselves orally [5,30].

A preterm newborn's physiological systems and oral tissues are immature, which delays the development of feeding abilities. They also lack the motor practice that would be provided by ingesting amniotic fluid during pregnancy. It has been demonstrated that standardized pre-feeding oral motor procedures, which involve stimulating oral structures and sucking on pacifiers, can increase feeding effectiveness, cut down on the time it takes to transition to full oral feeding and shorten hospital stays [31]. In the NICU, nutrition is a functional activity with a high priority that should be supported by the physiotherapist and other caregivers. Nutrition plays a significant role in the growth and development of the preterm baby [32].

The non-nutritive sucking strategy, which involves placing a pacifier or gloved finger in the mouth to stimulate the face muscles and intraoral structures, encourages sucking activity. Supporting the baby's cheeks from the outside and elevating the baby's head in semiflexion while feeding are other techniques used to facilitate sucking and swallowing [33]. According to a study in the literature, non-nutritive pacifier sucking before and after feeding with a catheter and bottle reduces the preterm baby's length of hospital stay, improves eating patterns, and decreases impulsive and defensive behaviors both before and after feeding [34].

Non-nutritive sucking techniques have a good impact and may help lower the death rate of premature infants. In order to enhance oral feeding practices in preterm infants, it is also advised that parents get involved in their care, develop and establish emotional connections with their children, and use low-cost, low-risk non-nutritive finger-sucking therapies [15].

Ghomi et al. established the beneficial effects of premature infant oral motor intervention (PIOMI) on preterm newborn oral eating in a randomized clinical trial. This study showed that early intervention in the NICU helped in early reflex maturation, thus having a positive impact on the health of neonates [5]. For preterm newborns in the NICU, oral feeding is frequently the last need for discharge and a major factor in prolonged hospital stays.

Primitive reflexes, which are also the basis for early movement and the stimulation of sensory organs and receptors, enable simple interactions with the environment. It is thought that this rise in sensory feedback and stimulation stimulates the development of functional connections and causes the expression of genes related to protein synthesis. Neuronal growth and connections are increased by stimulating glial cell proliferation [35]. As neurons grow larger, denser, and more connected, they eventually inhibit lower or more primitive areas of the brain via propriospinal projections. They will promote the development and activation of higher, more complex brainstem and neocortex regions, preventing long-term complications. Early combination rehabilitation can help preterm infants have better short-term clinical outcomes. Respiratory and neuromuscular motor development can be aided by early combined rehabilitative intervention [18].

## Conclusions

This review presents a better understanding of the maturation of survival reflexes and primitive reflexes and provides further insight into how a physiotherapist can concentrate on the early identification and development of these reflexes to prevent further complications. Assessing the primitive reflex in the NICU will help in the early identification of developmental delay and further help us predict reflex maturation. Promoting them will provide positive outcomes in terms of neonatal development. These reflexes improve the survival chances and thus reduce the hospital stay, opening up a door to a better quality of life for the growing infant. These reviews open up the scope for further studies to be done to promote survival reflexes in the NICU, along with a better insight into the physiotherapist’s role.
